# Interaction between CETP polymorphism and dietary insulin index and load in relation to cardiovascular risk factors in diabetic adults

**DOI:** 10.1038/s41598-021-95359-y

**Published:** 2021-08-05

**Authors:** Faezeh Abaj, Masoumeh Rafiee, Fariba Koohdani

**Affiliations:** 1 Department of Community Nutrition, School of Nutritional Sciences and Dietetics, Tehran University of Medical Sciences, Tehran, Iran; 2 Department of Clinical Nutrition, School of Nutrition and Food Science, Isfahan University of Medical Sciences (IUMS), Isfahan, Iran; 3 Department of Cellular, Molecular Nutrition, School of Nutritional Sciences and Dietetics, Tehran University of Medical Sciences (TUMS), PO Box: 141556117, Tehran, Iran

**Keywords:** Genetic interaction, Medical genetics, Cardiovascular diseases, Endocrine system and metabolic diseases

## Abstract

Gene–diet interactions may play an important role in the inter individual diversity observed in on cardiovascular disease (CVD) risk factors. Therefore, in the current study, we examined the interaction of CETP TaqB1 polymorphism with dietary insulin index and load (DII and DIL), in altering on CVD risk factors among type 2 diabetes mellitus (T2DM). In this cross-sectional study, blood samples were collected from 220 type 2 diabetic patients (134 females and 86 male) with a mean age of 52.24 years in Tehran, Iran. DIL and DII were obtained via validated food-frequency questionnaire (FFQ). Taq1B polymorphism was genotyped by the polymerase chain reaction-restriction fragment length polymorphism (PCR-RFLP) method. Biochemical markers including total cholesterol (TC), low-density lipoprotein (LDL), high-density lipoprotein (HDL), triglyceride (TG), superoxide dismutase (SOD), C-reactive protein (CRP), total antioxidant capacity (TAC), pentraxin-3 (PTX3), isoprostaneF2α (PGF2α). interleukin 18 (IL18), leptin and ghrelin were measured by standard protocol. Patients with B1B1 genotype had lower lipid profiles include LDL/HDL (P < 0.001) and TG (P = 0.04) when they consumed diets higher on the DIL and DII index. Moreover, carriers of B2B2 genotype who were in the last tertile of DIL had higher antioxidant and inflammatory markers include SOD (P = 0.01), PGF2α (P = 0.04) and CRP (P = 0.02). Further, a significant interaction between CETP TaqB1 and DII was shown in terms of WC (P = 0.01), where the highest WC were observed in B2B2 genotype carriers following a DII score. However, the highest inflammatory and antioxidant markers include CRP (P = 0.04), TAC (P = 0.01), SOD (P = 0.02), and PGF2α (P = 0.02) were observed in B2B2 genotype carriers when they consumed diets higher on the DII index. Based on the current study, it could be proposed that CETP polymorphism may be associated with CVD risk factors in T2DM patients with high following insulin indices, including DII and DIL. It seems that CETP Taq1B polymorphism can invert the result produced by insulin. This conclusion illustrates that the CETP Taq1B B1 allele could counteract the CVD risk induced by high DII and DIL.

## Introduction

Type 2 diabetes mellitus (T2DM) is a metabolic disorder that imposes an enormous burden on public health, which raise to 700 million by 2045^1^. Patients with T2DM should be considered at high risk of CVD^2^. In this way, the mortality rate in patients with T2DM two-to-four-fold increased risk versus non-CVD patients^2^. As a multifactorial condition, T2DM and CVD are also determined by environmental conditions such as dietary intake and genetic variations, which have been related to 50% of incidence^3^.

Recently, emerging data suggest that one of the main genetic targets is the cholesteryl ester transfer protein (CETP), which plays a crucial role in regulating lipid metabolism^4^. CETP is involved in the reverse cholesterol transport process by mediating the exchange of cholesteryl esters (CEs) and TGs from HDL to LDL in peripheral tissues to the hepatocytes^5^. CETP activity causes decrease and increase in serum levels of HDL-C and LDL-C, respectively, which may increase the risk of CVD^6^. The CETP gene is very polymorphic in humans, such that rs708272 (also called Taq1B) has been widely studied among CETP polymorphisms^7^. According to various studies, there is a relationship between rs708272 and various CVD risk factors including T2DM, hypertension, dyslipidemia, and low HDL, although the evidence is controversial: other studies have indicated a lack of such associations^7–9^.

On the other hand, one of the most important of environmental factors is diet, which play a major role in development of T2DM and CVD^10^. It seems that foods can induce postprandial insulin secretion and affect the management of hyperlipidemia, CVD, obesity, and DM^11^. With regards to the hypothesis of insulinogenic effects of the foods on the progression of the CVD, insulin indices including DII and DIL have been proposed^12^. DII and DIL can more accurately predict insulin response to overall food consumption compare to other indices. Importantly, DII and DIL could directly estimate the quantify and qualify of insulin secretion in response to the carbohydrate and protein rich foods, certain amino acids, and fatty acids^13,14^. Relatively few studies in the literature have investigated the correlation between DII, DIL, and risk of diabetes and CVD, and the correlation remains unclear within current nutrition research^15,16^.

Several studies which showed inconsistent results may be explained by the mixed effects of the SNP genetic and lifestyle factors (e.g. DII and DIL dietary) in lipid and glycemic metabolism, may play a main role in changes in metabolic condition^17^. Therefore, the interaction between genetics and diet in terms of nutrigenetic approach is essential in assessing these associations, which are still not well understood^18^. Some findings revealed that the CETP polymorphism interacts with dietary carbohydrate intake on metabolic factors, such as hypertension, dyslipidemia, obesity, insulin resistance (IR) and DM^19^. In this regard, several studies proposed a potential interaction between CETP SNP and dietary fat on plasma lipid and lipoprotein concentrations^20–22^. However, the number of researchers did not show that the TaqIB polymorphism in the CETP gene can affect cardio-metabolic responses to dietary intakes^23–25^.

To the authors’ knowledge, there has been no previous evaluating the interaction between the DII, DIL and CETP polymorphism towards CVD risk factors**.** Gene–diet interactions may play an important role in the inter individual diversity observed in anthropometric variables, inflammatory and antioxidant markers, serum levels of lipid and lipoproteins and as a result on CVD risk. Therefore, in the current study, we examined the interaction of CETP TaqB1 with dietary insulin index and load, in altering on cardiovascular risk factors including obesity indices (WC and BMI), lipid profiles (TG, HDL, LDL/HDL), inflammatory markers (IL-18, CRP, and PGF2α), and antioxidant markers (TAC and SOD).

## Results

### General characteristics and dietary intake according to CETP rs708272 genotype

In the current study, 220 patients with T2DM were evaluated. The genotype distributions were within HWE (P-value > 0.05). Table 1 shows the general characteristics based on each participant’s genotype. No significant difference was identified in the age, anthropometric data, physical activity and dietary intake between the three genotype groups.

**Table 1:** General characteristics and dietary intake according to *CETP* rs*708272* genotype.

	CETP (*rs708272*)			P value
	B1B1	B1B2	B2B2	
Age (year)	53.05 ± 4.92	52.51 ± 6.64	50.78 ± 6.17	0.26^a^
Weight, kg	77.45 ± 21.62	76.51 ± 14.62	75.44 ± 12.14	0.87^a^
Height, cm	161.05 ± 10.68	161.74 ± 9.76	161.84 ± 8.95	0.95^a^
BMI, kg/m^2^	29.52 ± 5.98	29.24 ± 5.05	28.84 ± 4.32	0.86^a^
WC, cm	90.57 ± 14.81	91.71 ± 12.19	91.81 ± 10.03	0.92^a^
Physical activity, MET	37.46 ± 5.47	38 ± 5.23	39 ± 5.45	0.47^a^
Total energy intake, kcal/day	2284.08 ± 572.5	2534.57 ± 870.01	2772.58 ± 1070.74	0.12^a^
Carbohydrate, g/day	297.98 ± 63.32	340.15 ± 117.61	349.98 ± 166.36	0.39^b^
Protein, g/day	78 ± 16.10	89.85 ± 32.95	90.20 ± 39.09	0.15^b^
Total fat, g/day	93.66 ± 41.91	99.07 ± 47.79	114.21 ± 53.29	0.21^b^
Saturated fatty acids, g/day	26 ± 9.32	26.86 ± 10.99	30.20 ± 13.71	0.28^b^
Monounsaturated fatty acids, g/day	32.16 ± 16.04	34.57 ± 18.76	39.44 ± 19.94	0.56^b^
Polyunsaturated fatty acids, g/day	22.77 ± 14.11	24.54 ± 14.86	28.44 ± 15.42	0.58^b^
Cholesterol, (mg/day)	216.56 ± 63.71	22.54 ± 185.77	239.53 ± 115.22	0.84^b^
Dietary fiber, g/day	34.74 ± 14.34	43.78 ± 22.27	42.64 ± 27.19	0.20^b^

Values are means ± SDs.

^a^ANOVA tests (crude model).

^b^ANCOVA tests (adjusted for energy).

### Association between baseline characteristic and metabolic markers with dietary insulin indices

A statistical analysis of the basic information of patients, among DIL and DII tertiles, are presented in Tables 2 and 3 respectively. Higher tertiles of DIL and DII was associated with higher DIL and DII score respectively (*P* = 0.001). Individuals following a DIL (*P* = 0.001) and DII (*P* = 0.006) was more likely to be male. Subjects with a higher DIL tertile presented greater energy intake values (*P* = 0.001). Patients in the highest tertile of DIL (*P* = 0.01) and DII (*P* = 0.01) were more likely to have lower ghrelin concentrations. The subjects with a higher tertile of DIL have more TG concentration (*P* = 0.04). There were no statically significant differences in terms of other variables (*P* > 0.05).

**Table 2:** The association between baseline characteristic and metabolic markers with dietary insulin load (DIL).

Metabolic markers (N)	Tertiles of DIL			*P*^a^
	T1	T2	T3	
Sex (Male) N% (220)	19 (22.1%)	26 (30.2%)	41 (47.7%)	**0.001**
Cigarette smoking (yes) N% (220)	17 (37.8%)	14 (31.1%)	14 (31.1%)	0.76
Alcohol consumption (yes) N% (220)	1 (25%)	2 (50%)	1 (25%)	0.78
Familial history of diabetes (yes) N% (220)	65 (34.6%)	64 (34%)	59 (31.4%)	0.35
Glucose-lowering medication (220) Metformin and glybenclamid N%	26 (28.3%)	37 (40.2%)	29 (31.5%)	0.19
Other medications N%	47 (36.7%)	37 (28.9%)	44 (34.4%)	
Age (year) (220)	52.41 ± 6.13	52.76 ± 6.7	51.53 ± 6.94	0.49
BMI (kg/m^2^) (220)	28.74 ± 5.3	29.96 ± 4.58	28.86 ± 5.06	0.15
WC (cm) (220)	90.37 ± 14.04	92.77 ± 10.44	91.74 ± 11.37	0.33
Physical activity (Met.wk) (220)	38.65 ± 6.01	38.39 ± 4.6	37.38 ± 5.16	0.31
Energy intake (kcal/day) (220)	1890.67 ± 399.49	2481.65 ± 497.49	3288.51 ± 1010.12	** < 0.001**
DIL score* (220)	77,060.59 ± 17,094.69	108,647.14 ± 16,732.69	177,050.8451 ± 104,959.09	** < 0.001**
DII score* (220)	40.20 ± 7.42	45.67 ± 7.59	57.46 ± 34.05	** < 0.001**
HDL-c (mg/dl) (220)	53.95 ± 10.15	52.5 ± 12.17	53.5 ± 11.78	0.54
LDL-c (mg/dl) (220)	120.46 ± 31.6	117.62 ± 32.11	117.53 ± 38.52	0.82
CH (mg/dl) (220)	215.2 ± 99	204.04 ± 59.71	213.36 ± 67.77	0.44
LDL/HDL (220)	2.28 ± 0.65	2.30 ± 0.64	4.79 ± 21.51	0.69
TG (mg/dl)* (220)	114.50 ± 136.75	123.5 ± 274.25	173 ± 136	**0.04**
Leptin (ng/ml) (172)	24.18 ± 15.03	22.55 ± 15.55	24.89 ± 16.8	0.93
Ghrelin (ng/ml) (172)	2.22 ± 1.08	2.55 ± 1.29	1.88 ± 0.73	**0.01**
hs.CRP (mg/L)* (159)	2.63 ± 2.35	2.86 ± 2.83	3.65 ± 1.90	0.08
PTX3 (ng/ml) (159)	2.67 ± 0.39	2.74 ± 0.44	2.81 ± 0.55	0.65
IL18 (pg/ml) (159)	244.24 ± 30.83	248.75 ± 28.03	252.56 ± 28.70	0.64
TAC (g/dl) (159)	2.55 ± 0.63	2.33 ± 0.46	2.34 ± 0.34	0.34
SOD (U/ml) (159)	0.14 ± 0.04	0.13 ± 0.03	0.15 ± 0.06	0.5
PGF2α (pg/ml) (159)	72.77 ± 6.32	75.73 ± 6.15	71.53 ± 8.09	0.25

Data are presented as mean ± standard deviation (SD) or percent or Median ± IQR* (TG, hs.CRP, DIL and DII).

DIL: dietary insulin load; DII: dietary insulin index; BMI: body mass index; HDL-c high density lipoprotein cholesterol; LDL-c: low density lipoprotein cholesterol; CH: cholesterol; TG: triglyceride; CRP: C-reactive protein; PTX3: Pentraxin 3; IL18: interleukin 18; TAC: total antioxidant capacity; SOD: superoxide dismutase; PGF2α: prostaglandinF2α.

^a^Obtained from ANOVA or Chi-square or Kruskal–Wallis test where appropriate.

Bold values denote statistical significance at the p < 0.05 level.

**Table 3:** The association between baseline characteristic and metabolic markers with dietary insulin index (DII).

Metabolic markers (N)	Tertiles of DII			*P*^a^
	T1	T2	T3	
Sex (male) (220)	18 (20.9%)	31 (36%)	37 (43%)	**0.006**
Cigarette smoking (yes) N% (220)	17 (37.8%)	13 (28.9%)	15 (33.3%)	0.64
Alcohol consumption (yes) N% (220)	1 (25%)	2 (50%)	1 (25%)	0.79
Familial history of diabetes (yes) N% (220)	62 (33%)	63 (33.5%)	63 (33.5%)	0.91
Glucose-lowering medication (220) Metformin and glybenclamid N%	28 (30.4%)	37 (40.2%)	27 (29.3%)	0.26
Other medications N%	44 (34.4%)	38(29.7%)	46 (35.9%)	
Age (year) (220)	52.72 ± 5.96	51.76 ± 6.95	52.25 ± 6.39	0.66
BMI (kg/m^2^) (220)	29.03 ± 4.55	29.86 ± 6.07	28.67 ± 4.12	0.46
WC (cm) (220)	90.81 ± 10.63	93.49 ± 14.4	90.54 ± 10.53	0.84
Physical activity (Met.wk) (220)	38.59 ± 6.2	38.12 ± 4.82	37.73 ± 4.78	0.62
Energy intake (kcal/day) (220)	2547.45 ± 879.53	2389.18 ± 744.25	2736.63 ± 1018.57	0.06
DIL* (220)	80,291.31 ± 38,739.74	102,185.87 ± 32,635.11	165,478.10 ± 113,442.12	** < 0.001**
DII* (220)	38.03 ± 5.38	45.57 ± 3.33	60.14 ± 29.11	** < 0.001**
HDL-c (mg/dl) (220)	53.43 ± 11.48	53.06 ± 10.69	53.46 ± 12.08	0.99
LDL-c (mg/dl) (220)	119.51 ± 33.64	115.37 ± 29.29	120.82 ± 38.97	0.76
CH (mg/dl) (220)	211.01 ± 62.89	218.98 ± 99.1	202.36 ± 63.4	0.54
LDL/HDL (220)	2.27 ± 0.6	2.24 ± 0.63	4.87 ± 21.50	0.29
TG (mg/dl)* (220)	114.50 ± 136.75	123.50 ± 274.25	173 ± 136	0.35
Leptin (ng/ml) (172)	22.33 ± 14.01	25.4 ± 17.03	23.64 ± 16.04	0.84
Ghrelin (ng/ml) (172)	1.99 ± 0.43	2.68 ± 1.4	1.86 ± 0.87	**0.01**
hs.CRP (mg/L)* (159)	2.63 ± 2.35	2.86 ± 2.83	3.65 ± 1.90	0.31
PTX3 (ng/ml) (159)	2.67 ± 0.39	2.68 ± 0.42	2.83 ± 0.52	0.51
IL18 (pg/ml) (159)	247.20 ± 32.45	244.87 ± 27.71	250.15 ± 28.71	0.86
TAC (g/dl) (159)	2.51 ± 0.58	2.4 ± 0.6	2.44 ± 0.41	0.78
SOD (U/ml) (159)	0.14 ± 0.03	0.13 ± 0.04	0.15 ± 0.05	0.29
PGF2α (pg/ml) (159)	73.61 ± 6.93	73.21 ± 5.87	72.38 ± 7.88	0.85

Data are presented as mean ± standard deviation (SD) or percent or Median ± IQR* (TG, hs.CRP, DIL and DII).

DIL: dietary insulin load; DII: dietary insulin index; BMI: body mass index; HDL-c high density lipoprotein cholesterol; LDL-c: low density lipoprotein cholesterol; CH: cholesterol; TG: triglyceride; CRP: C-reactive protein; PTX3: Pentraxin 3; IL18: interleukin 18; TAC: total antioxidant capacity; SOD: superoxide dismutase; PGF2α: prostaglandinF2α .^a^: Obtained from ANOVA.

Bold values denote statistical significance at the p < 0.05 level.

### Interaction between CETP Taq1B polymorphism and dietary insulin indices on cardiovascular risk factors

Tables 4 and 5 show the interactions between CETP TaqB1 polymorphism and DIL and DII on anthropometric indices and several biochemical markers.

**Table 4:** Mean values of CVD risk factors across CETP genotypes (B1B, B1B2, B2B2) based on low and high DIL intake.

Variables	DIL	Mean ± SD			P_1_	P_2_
		B1B1	B1B2	B2B2	Model (1)	Model (2)
WC (cm)					0.07	**0.04**
	T_1_	93.51 ± 3.95	91.75 ± 1.66	88.50 ± 3.28		
	T_2_	95.33 ± 4.84	92.30 ± 1.59	93.53 ± 3.28		
	T_3_	95.5 ± 5.92	91.06 ± 1.61	94.53 ± 3.16		
TG (mg/dl)					**0.03**	**0.04**
	T_1_	349.83 ± 46.92	164.86 ± 16.25	150.46 ± 31.87		
	T_2_	223.25 ± 57.46	195.15 ± 15.93	169.69 ± 31.87		
	T_3_	145.88 ± 38.13	182.09 ± 15.78	196.86 ± 29.67		
HDL (mg/dl)					**0.03**	**0.01**
	T_1_	51.66 ± 4.47	53.64 ± 1.53	54.33 ± 2.83		
	T_2_	56.88 ± 3.65	50.21 ± 1.47	53.53 ± 3.04		
	T_3_	58.50 ± 5.48	52.90 ± 1.49	55.15 ± 3.04		
LDL/HDL					** < 0.001**	** < 0.001**
	T_1_	4.89 ± 5.34	2.34 ± 1.49	2.20 ± 2.96		
	T_2_	2.20 ± 4.36	2.38 ± 1.44	2.02 ± 2.96		
	T_3_	2.08 ± 3.56	2.22 ± 1.45	2.29 ± 2.76		
CRP (mg/l)					**0.02**	**0.02**
	T_1_	1.71 ± 0.45	2.37 ± 0.29	2.67 ± 0.64		
	T_2_	3.23 ± 1.28	2.15 ± 0.38	3.43 ± 1.28		
	T_3_	4.69 ± 0.74	2.88 ± 0.38	5.43 ± 1.28		
IL18(pg/ml)					**0.02**	0.06
	T_1_	253.85 ± 9.47	243.72 ± 6.15	227.47 ± 13.40		
	T_2_	236.43 ± 26.81	244.69 ± 8.08	238.56 ± 15.48		
	T_3_	284.34 ± 26.81	253.49 ± 8.08	305.80 ± 16.81		
SOD (U/ml)					**0.008**	**0.01**
	T_1_	0.14 ± 0.01	0.14 ± 0.009	0.11 ± 0.04		
	T_2_	0.10 ± 0.04	0.13 ± 0.01	0.14 ± 0.02		
	T_3_	0.07 ± 0.04	0.14 ± 0.01	0.21 ± 0.02		
PGF2alpha (pg/ml)					0.06	**0.02**
	T_1_	74.62 ± 2.24	71.89 ± 1.45	61 ± 6.36		
	T_2_	75 ± 6.36	75.86 ± 1.91	74.66 ± 3.67		
	T_3_	73.25 ± 3.18	71.63 ± 1.91	75 ± 6.36		

Mean values of cardiovascular risk factors across CETP genotypes (B1B1, B1B2, B2B2) based on tertiles of DIL intake.

P1: P-value for curd model; P2: P-value for the adjusted model by age, gender, physical activity, smoking, alcohol consumption, and familial history of diabetes.

Bold values denote statistical significance at the p < 0.05 level.

**Table 5:** Mean values of CVD risk factors across CETP genotypes (B1B, B1B2, B2B2) based on low and high DII intake.

Variables	DII	Mean ± SD			P_1_	P_2_
		B1B1	B1B2	B2B2	Model (1)	Model (2)
BMI (kg/m^2^)					0.20	0.08
	T_1_	29.39 ± 1.31	29.16 ± 0.72	28.10 ± 1.41		
	T_2_	28.10 ± 1.35	30.19 ± 0.64	31.23 ± 2.83		
	T_3_	29.01 ± 1.35	28.32 ± 0.65	32.48 ± 2.45		
WC (cm)					**0.02**	**0.01**
	T_1_	93.33 ± 3.35	90.50 ± 1.71	86.34 ± 3.22		
	T_2_	95.5 ± 6.71	94.91 ± 1.52	94.85 ± 3.10		
	T_3_	95.5 ± 5.81	89.39 ± 1.55	97 ± 3.22		
TG (mg/dl)					**0.02**	**0.02**
	T_1_	486 ± 65.26	185.82 ± 16.85	159.35 ± 30.21		
	T_2_	223.25 ± 56.51	192.78 ± 15.10	191.64 ± 30.21		
	T_3_	162.83 ± 32.63	164.51 ± 15.38	169.30 ± 31.35		
LDL (mg/dl)					0.08	0.08
	T_1_	143.75 ± 16.77	117.5 ± 4.94	131.57 ± 8.96		
	T_2_	114.33 ± 19.37	116.91 ± 4.40	118.21 ± 8.96		
	T_3_	113.16 ± 9.68	119.82 ± 4.48	109.07 ± 9.30		
LDL/HDL					** < 0.001**	** < 0.001**
	T_1_	4.89 ± 5.34	2.28 ± 1.57	2.37 ± 2.85		
	T_2_	2.14 ± 6.17	2.33 ± 1.40	1.88 ± 2.85		
	T_3_	2.13 ± 3.08	2.33 ± 1.42	2.28 ± 2.96		
CRP (mg/l)					**0.008**	**0.04**
	T_1_	1.71 ± 0.45	2.22 ± 0.36	2.12 ± 0.73		
	T_2_	2.43 ± 1.27	2.58 ± 0.33	3.87 ± 0.90		
	T_3_	3.23 ± 1.27	2.50 ± 0.34	4.69 ± 0.73		
TAC (g/dl)					**0.02**	**0.01**
	T_1_	2.76 ± 0.17	2.43 ± 0.14	2 ± 0.35		
	T_2_	2.1 ± 0.49	2.31 ± 0.12	2.56 ± 0.28		
	T_3_	2.5 ± 0.49	2.41 ± 0.13	2.93 ± 0.28		
SOD (U/ml)					**0.001**	**0.02**
	T_1_	0.14 ± 0.01	0.15 ± 0.01	0.10 ± 0.02		
	T_2_	0.10 ± 0.03	0.13 ± 0.01	0.16 ± 0.02		
	T_3_	0.07 ± 0.03	0.15 ± 0.01	0.21 ± 0.02		
PGF2alpha (pg/ml)					0.05	**0.02**
	T_1_	74.62 ± 2.26	72.12 ± 1.85	70.33 ± 3.70		
	T_2_	75 ± 6.41	73.66 ± 1.65	74.66 ± 3.70		
	T_3_	75.5 ± 6.41	72.71 ± 1.71	78. 5 ± 4.53		

Mean values of cardiovascular risk factors across CETP genotypes (B1B1, B1B2, B2B2) based on tertiles of DII intake.

P1: P-value for curd model; P2: P-value for the adjusted model by age, gender, physical activity, smoking, alcohol consumption, and familial history of diabetes.

Bold values denote statistical significance at the p < 0.05 level.

Interactions between CETP TaqB1 polymorphism and DIL were significant in terms of WC (P_1_-interaction = 0.07, P_2_-interaction = 0.04) after adjusting potential confounders: carriers of B2B2 genotype who were in the last tertile of DIL had higher WC. Besides, CETP TaqB1 polymorphism and DIL interactions were significant in terms of lipid markers include LDL/HDL (P_1_-interaction < 0.001, P_2_-interaction < 0.001), TG (P_1_-interaction = 0.03, P_2_-interaction = 0.04), and HDL (P_1_-interaction = 0.03, P_2_-interaction = 0.01) in both crude and adjustment model: carriers of the B1B1 genotype had lower LDL/HDL, TG, and higher HDL when they consumed diets higher on the DIL index.

Also, CETP TaqB1 polymorphism and DIL interactions were significant in terms of antioxidant and inflammatory markers include SOD (P_1_-interaction = 0.008, P_2_-interaction = 0.01), PGF2α (P_1_-interaction = 0.06, P_2_-interaction = 0.02) and CRP (P_1_-interaction = 0.02, P_2_-interaction = 0.02); higher SOD, PGF2α and CRP, values were observed in B2B2 genotype carriers when they consumed diets higher on the DIL index. Although IL-18 was significant in the crude model (P = 0.02), after adjusting for potential confounders including age, gender, physical activity, smoking, alcohol consumption, and familial history of diabetes, this significance was disappeared (p = 0.06) (Table 4).

Further, a significant interaction between CETP TaqB1 and DII was shown in terms of WC (P_1_-interaction = 0.02, P_2_-interaction = 0.01), where the highest WC were observed in B2B2 genotype carriers following a DII score. A significant interaction between CETP rs708272 and DII was shown in terms of lipid markers include TG (P_1_-interaction = 0.02, P_2_-interaction = 0.02) and LDL/HDL (P_1_-interaction < 0.001, P_2_-interaction < 0.001): B1B1 homozygotes in the highest tertile of DII had lower TG concentration and LDL/HDL ratioAlso, a significant interaction between CETP TaqB1 and DII was shown in terms of antioxidant and inflammatory markers CRP (P_1_-interaction = 0.008, P_2_-interaction = 0.04) TAC (P_1_-interaction = 0.02, P_2_-interaction = 0.01), SOD (P_1_-interaction = 0.001, P_2_-interaction = 0.02), and PGF2α (P_1_-interaction = 0.05, P_2_-interaction = 0.02): individuals with B2B2 genotype in the last tertile of DII had higher CRP, TAC, SOD, and PGF2α (Table 5).

## Discussion

The key findings of the current study were the significant interaction result of CETP rs708272 polymorphism with DIL and DII on obesity indices (WC and BMI), lipid profiles (TG, HDL, LDL/HDL), inflammatory markers (IL-18, CRP, and PGF2α), and antioxidant markers (TAC and SOD) in T2DM patients.

In the present study, B2B2 genotype carriers in the last tertile of DII and DIL were have greater WC as a central obesity marker. CETP Taq1B polymorphism was found to be able to increase the association between dietary insulin indices and obesity. Although several studies have reported an association between DIL and DII scores and obesity^26,27^, the interaction between DII and DIL with CETP polymorphism on obesity was not evaluated yet. A high score of DIL and DII may lead to greater body fat formation by promoting pre-adipocytes differentiation and proliferation to adipocytes^26,27^. Besides, a higher insulin indices score may cause reductions in insulin sensitivity and lipolysis and body fat accumulation by increasing insulin growth factor-1 (IGF-1)^28–30^. Additionally, CETP polymorphisms was associated with an elevated risk of obesity and obesity-related diseases^31–33^. Although plasma CETP activity has been reported to be elevated in obese subjects^34–38^, this elevation is eliminated with the development of T2DM^35,39,40^. Experimental studies reported that expression of CETP in a diabetic obese animal that cannot normally express CETP, prevented the development of atherosclerotic lesions when they consumed diet-induced obesity^41^. Although, B2 allele of this polymorphism is associated with decreased CETP activity^42,43^ and CETP concentration^44,45^. Moreover, previous studies, that suggested there is an inverse association between plasma glucose and CETP activity in T2DM patients^46,47^. Therefore, in our study diabetic patients carriers, B2 allele with reduced CETP activity who consume a diet with higher insulin index which might be associated with higher plasma glucose may act synergistically to elevate a patient’s susceptibility to impaired postprandial metabolism, which relates to obesity^48^.

The present results also demonstrated the highest inflammatory factors (CRP and PGF2α) in the B2B2 genotype carriers who follow a DIL score and DII. As indicated in the findings, there was a significant interaction between Taq1B CETP polymorphism and DII/DIL in association with anthropometric indices. Numerous findings have reported obesity as causing chronic low-grade inflammatory disorder, contributing to the progression of T2DM and CVD^49^. In these conditions, human adipose tissue secretes a high level of inflammatory markers, including IL-18, CRP, and PGF2α^50^. A further novel finding is Taq1B polymorphism was able to inverse the association of DII and DIL and oxidant status, so that the highest TAC and SOD was observed in the B2B2 genotype following a DIL and DII. However, there is no available study about the relation between DIL/DII and CETP polymorphism interaction with antioxidant status, several studies have reported that insulin concentrations and insulin resistance lead to an imbalance between oxidant and antioxidant systems, a condition known as oxidative stress^51^. In recent years, oxidative stress has been implicated in T2DM pathogenesis via reduced antioxidant enzyme activity, such as SOD and TAC^52^. It seems that CETP Taq1B polymorphism can invert the result produced by insulin.

Finally, another promising finding was the significant interaction between Taq1B polymorphism and DII/DIL on lipid profile markers, including TG, HDL, and LDL/HDL. The lowest TG and LDL/HDL, and the highest HDL, were observed in the B1B1 genotype carriers following a DIL and DII. In line with our study, systematic reviews showed that the B1B1 genotype is associated with a better response to nutritional interventions, compared with carriers of B2 alleles^53^. There has been limited study of interaction between Taq1B polymorphism and dietary intake. In this regard, Gammon et al. has showed a significant interaction, where B1B1 homozygotes had a lower TG/HDL ratio after a kiwifruit intervention, compared to a control diet, while B2 carriers were not affected^54^. Nahid Ramezani-Jolfaie et al. revealed that, for diabetic patients, dietary oil treatments would be more helpful (lower LDL; HDL, TG; HDL, TC; HDL, Insulin, and HOMA-IR) among subjects with B1B1 alleles than among B2 allele carriers^55^. Some authors have also suggested that subjects with B1B1 allele of CETP polymorphism showed a better response in regards to high carbohydrate dietary interventions. Overall these findings are in accordance with findings reported by Juan Dua et al., demonstrating that males with CETP Taq1B B1B1 allele have higher apo A-I and HDL concentrations after following a high carbohydrate and low fat (HC/LF) diet for 6 days^22^. Besides, Perez et al. reported that carriers of B2 alleles who consumed sucrose as more than 5% of total kcal/day had higher TC and LDL serum levels, compared to B1B1 homozygotes^56^. A series of recent studies have indicated that plasma CETP activity decreased by hyperinsulinemia condition in healthy subjects, but not in diabetic patients^57^. Siewert et al. suggested that insulin has a direct influence on CETP, but in IR conditions, this particular insulin action may be diminished^58^. Therefore, it is suggested that diabetic patients with B1B1 allele may be counteracted with CETP activity and HDL plasma reduction.

### Limitation and strength

Limitations of the present study including the cross-sectional design, so any causality cannot be argued; the use of FFQ for dietary assessing, which may have resulted in memory bias; small sample size, which may have led to weak statistical to determine significant results. Furthermore, our participants were from the Iranian country which may not be generalized due to racial and regional differences (52). Due to financial limitations, it was not possible to perform western blot analysis to determine whether rs708272 SNP alters the expression of CETP. Despite the limitations mentioned above, this is the first effort to study the interaction between CETP rs708272 polymorphism and dietary insulin indices on cardio-metabolic risk factors. Recognition of these gene-diet interactions could be determining in prescribe personalized nutritional recommendations for the improvement and management of CVD risk in T2DM patients. Finally, these results can be used in combination with a patient’s genetic history to provide more applicable and tailored nutritional advice for preventing or attenuating cardiovascular disease in T2DM patients.

## Conclusion

Based on the current study, it could be proposed that CETP polymorphism may be associated with CVD risk factors in T2DM patients with high following insulin indices, including DII and DIL. This conclusion illustrates that the CETP Taq1B B1 allele could counteract the CVD risk induced by high DII and DIL. This could be critical for clinical diagnosis and gene-based therapy. Due to the limited nature of the study, further research with larger sample size is warranted to assess the results on other populations.

## Methods

### Study population

A cross-sectional study was designed with 220 diabetic patients in Tehran, Iran, who had participated in a large study conducted previously^59^. Diabetic patients who had fasting blood sugar > 126 mg/dl or were using glucose-lowering medications without a history of inflammatory diseases, CVD, stroke, and cancers were included in the study. Besides, patients who were pregnant, addict, taking anti-inflammatory medications and using insulin, lactating patients, and also their total calorie intake was not in range between 800 and 4200 were excluded. All study subjects gave their written informed consent and the study was conducted based on the Declaration of Helsinki, and Ethics Committee of the Tehran University of Medical Sciences approved the protocol (no. 15060).

### General, anthropometric and physical activity assessments

Information such as age, disease history, and medication was collected from each participant by questionnaire. Anthropometric measurements, including height (m) and weight (kg) were evaluated without shoes, and using a digital scale; waist circumference (WC) was evaluated at the narrowest part of the abdomen. Finally, body mass index (BMI) was computed by dividing weight (kg) by height squared (meters). Physical activity was measured by the short-form International Physical Activity Questionnaire (IPAQ)^60^.

### Biochemical assessments

After fasting for 12–14 h, all blood samples were obtained and centrifuged for 10 min at 3000 rpm to extract serums, which were then aliquoted into 1 ml tubes and refrigerated at 70 °C until analysis. The samples were tested using an auto-analyzer BT 1500 (Selectra 2; Vital Scientific, Spankeren, Netherlands). The levels of TG and TC in the blood were determined using an enzymatic technique and commercially available kits (Pars Azmoon, Iran). Serum levels of HDL-C and LDL-C were determined by turbidimetry on a Roche Hitachi analyzer (Roche, Germany). The serum levels of leptin and ghrelin were also determined using the ELISA method (Bioassay Technology Co, China and Mediagnost, Germany, respectively). The ELISA method was used to determine the amounts of inflammatory markers in the blood, such as IL-18 and PTX3 (Shanghai Crystal Day Biotech Co., Ltd). The IL-18 ELISA kit had a sensitivity of 28 ng/l, and the intra-assay and interassay coefficients of variation (CV) were less than 10% and 12%, respectively. The PTX3 ELISA kit has a sensitivity of 0.05 ng/ml, and the intra-assay and interassay CVs were less than 10% and 12%, respectively. ELISA was used to assess hs-CRP levels in the blood (Diagnostic Biochem Canada Inc., London, Ontario, Canada). The intra-assay and interassay CVs were both less than 5% and 9.5%, respectively. Spectrophometry was used to determine the serum's total antioxidant capacity (TAC). The colorimetric approach was used to determine the serum enzymatic activity of superoxide dismutase (SOD) (Cayman Chemical Company, USA). ELISA (Shanghai Crystal Day Biot) was used to determine the concentration of 8-isoprostane F2 in the blood. All assessment was conducted at the Nutrition and Genomics Laboratory at TUMS.

### Dietary assessment

A validated 147-item food-frequency questionnaire (FFQ) was used to obtain a common dietary intake for patients^61^. The FFQ contained 147 food items with standard portion sizes commonly used by Iranians, based upon on measurements widely used in the community (e.g. number of slices for bread, glasses for drinks, plates for rice, etc.)^62^. The portion sizes in the FFQ were converted into amounts of foods consumed. An expert interviewer carried out the FFQ and recorded participant responses. All reported consumption frequencies were converted to grams per day using household measures. The daily consumption of nutrients and total energy were calculated for each subject by using the Iranian food composition table (FCT) and the United States Department of Agriculture (USDA) guidelines. Validity and reliability of the FFQ have been confirmed previously^63^.

### Assessment of DIL and DII

DII was based on prior studies conducted by Brand-Miller^64^. DII calculates the incremental insulin area under the curve over 2 h, in response to the consumption of a 1000-kJ portion of the test food, then divided by the area under the curve after ingestion of a 1000-kJ portion of the reference food^14,64,65^. In the current study, food items in the FFQ and the Brand-Miller study were matched in terms of energy content, carbohydrates, fiber, protein, and fat. To evaluate the average DIL, the insulin load from each food over the past year was calculated by the formula:

$${\text{Insulin load of food }} = {\text{ Insulin index of food }} \times {\text{ energy content of food }}\left( {{\text{kcal}}/{\text{day}}} \right)$$

By summing up the insulin load from each food, DIL was determined. The DII of each food was computed by dividing DIL by total energy intake.

### Genotyping

DNA genotyping was carried out by the salting-out extraction method, as previously published^66^. CETP polymorphism (rs708272) was genotyped by the polymerase chain reaction-restriction fragment length polymorphism (PCR-RFLP) method. The PCR technique was employed by using primer (F:50-CACTAGCCCAGAGAGAGGAGTG-30; R: 50-TGAGCCCAGCCGCACACTAAC-30). 2% agarose gel electrophoresis was used to analyze the product.

### Statistical analysis

All data were analyzed using IBM SPSS Statistics (version 25; SPSS Inc., IL). Significance level was considered P < 0.05, and normality was analyzed by Kolmogorov Smirnov test.

Adherence to Hardy–Weinberg equilibrium (HWE) was determined by using the chi-square test. The means of variables across the tertiles of DII and DIL were expressed as means ± SDs. Crude means between three genotypes (B1B1, B1B2, and B2B2) groups were compared using a one-way ANOVA test. The interaction between CETP rs708272 polymorphism and insulin indices (DII and DIL) on CVD risk factor (BMI, WC, HDL, LDL, LDL/HDL, TC, TG, CRP, IL-18, TAC, SOD, PGF2α) was performed by a generalized linear regression model (GLM) in both the crude and adjusted models. In all interaction analyses (DIL and DII), age, gender, physical activity, smoking, alcohol consumption, and familial history of diabetes were matched in the adjusted model. Further, all analysis based on DIL was adjusted to energy intake.

## Data Availability

The data are not publicly available due to containing private information of participants. Data are however available from the authors upon reasonable request and with permission of Fariba Koohdani.
